# Community Reserves: Their significance for the conservation of mammals in a mosaic of community-managed lands in Meghalaya, Northeast India

**DOI:** 10.1371/journal.pone.0280994

**Published:** 2023-01-26

**Authors:** Adrian Wansaindor Lyngdoh, Honnavalli N. Kumara, Santhanakrishnan Babu, P. V. Karunakaran

**Affiliations:** 1 Sálim Ali Centre for Ornithology and Natural History, Coimbatore, Tamil Nadu, India; 2 Forests & Environment Department, Shillong, Meghalaya, India; Centre for Cellular and Molecular Biology, INDIA

## Abstract

Community Reserves (CRs) have been advocated for increasing the protected area coverage in northeast India where the land is primarily owned and managed by local indigenous institutions. To understand the significance of these reserves for the conservation of mammals, we investigated the diversity and abundance of mammals in five CRs in the Khasi Hills of Meghalaya as well as interviewed 75 local villagers to assess the hunting practices and perceptions of the Indigenous Khasis on mammals. We employed 60 camera traps in the CRs and undertook a recce survey (day-time and night-time) for capturing the diversity in the CRs. We used photo-capture rate and encounter rate as indices of relative abundance in the CRs. We used an exact multinomial test to test differences of opinion among the respondents of the five CRs. We found a relatively low abundance of mammals in the CRs, yet they persist. A total of 28 species were detected through camera trapping and recce survey and an additional 12 species were reported by respondents to also occur in the CRs. Among the respondents, it was believed that the decline in mammal populations was largely driven by habitat loss and degradation (82.67%) while only a few believed it was also driven by hunting (5.33%). Respondents also believed that the presence of the reserves and awareness programs taken under them had also led to a reduction in hunting (20%) in their area. Although, some attributed it to a general decline in wildlife populations and forest cover (21.33%). Thus, despite these CRs being small (<2 km^2^) and isolated, they still harbour mammal species and are important for retaining remnant forest patches in a landscape that is highly fragmented.

## Introduction

Human activities have led to habitat loss, fragmentation, and degradation, with the remaining natural vegetation in biodiversity hotspots covering only 4.6% of the earth’s surface [1]. These human impacts are especially influential at regional scales, having a detrimental effect on terrestrial biodiversity, causing long-term land cover change and catalysing the extinction of many species [2]. Without any conservation intervention to cap the rate of habitat loss, this could further lead to the extinction of half of the threatened species in most of the major biodiversity hotspots in the world [3].

India lost about 28% of its original forests since 1930’s, where northeast India being one of the regions where the loss was most concentrated [4]. Large-scale deforestation in the region—driven by the conversion of forests to human land-uses such as settled agriculture, horticultural plantations, settlements, illegal logging, mining, and *Jhum* cultivation (shifting cultivation)—has led to extensive fragmentation, and degradation of primary natural habitats [5–7]. Such degradation of natural habitats has led to a decline in populations of many mammal species in northeast India [8–14] as well as an increase in human-wildlife conflict leading to retaliatory killings [9, 15].

Hunting of wild animals is widely practiced in the tropics for a variety of reasons including subsistence, traditional practice, management of pests or trade [16]. Hunters usually prefer large-bodied species but they would capture and kill any animal they encountered, this has led to the extirpation of local populations of vulnerable species as well as the alteration of ecosystem function [16]. Increased accessibility to forests and increasing demand of wildlife products has further exacerbated its unsustainability [17]. Over one-third of the indigenous population in India are from the north-eastern region, traditionally dependent on meat and most engaging in hunting as part of their age-old socio-cultural practices [18]. In recent times, however, hunting of wildlife has intensified to meet the demands of wildlife trade [18]. Hunting is practiced for consumption of meat, medicinal use, recreation and commercial purposes [9, 17, 19, 20]. This has led to the decline of many mammal species in the region e.g., [11, 15]. Although hunting of wildlife is prohibited under the country’s law, enforcement on the ground is weak or lacking [21] and hunting may still occur even within the protected areas [22, 23]. Nevertheless, protected areas (PAs) are still invaluable for attenuating the impact of hunting for the continued persistence of mammal species [24].

The land in northeast India is primarily owned and managed by local people and traditional institutions [25], thus, Community Conservation Reserves (or ‘Community Reserves’) were encouraged. There are about 205 of such reserves spread across the region covering an area of about 1148.74 km^2^ (National Wildlife Database, Wildlife Institute of India). Community Reserves (CRs) are PAs primarily managed by indigenous communities, that can also aid in reducing exploitation of wildlife [21]. Despite being smaller in size, many CRs have low human pressure and can help in providing crucial habitats in human-dominated landscapes and in maintaining biodiversity values [26–29].

In this study, we aimed to investigate the diversity and abundance of mammals across five CRs of Ri Bhoi district, Meghalaya using camera trapping, reconnaissance survey and semi-structured questionnaire surveys. We used semi-structured questionnaire survey to investigate the perceptions of the indigenous Khasi community on trends in the population status of mammals in their area, trends in human-wildlife conflict, their hunting practices and wild meat consumption preferences. The target study group were primarily the agrarian indigenous Khasi community residing in rural areas surrounding these CRs.

## Materials and methods

### Study area

**Fig 1:**
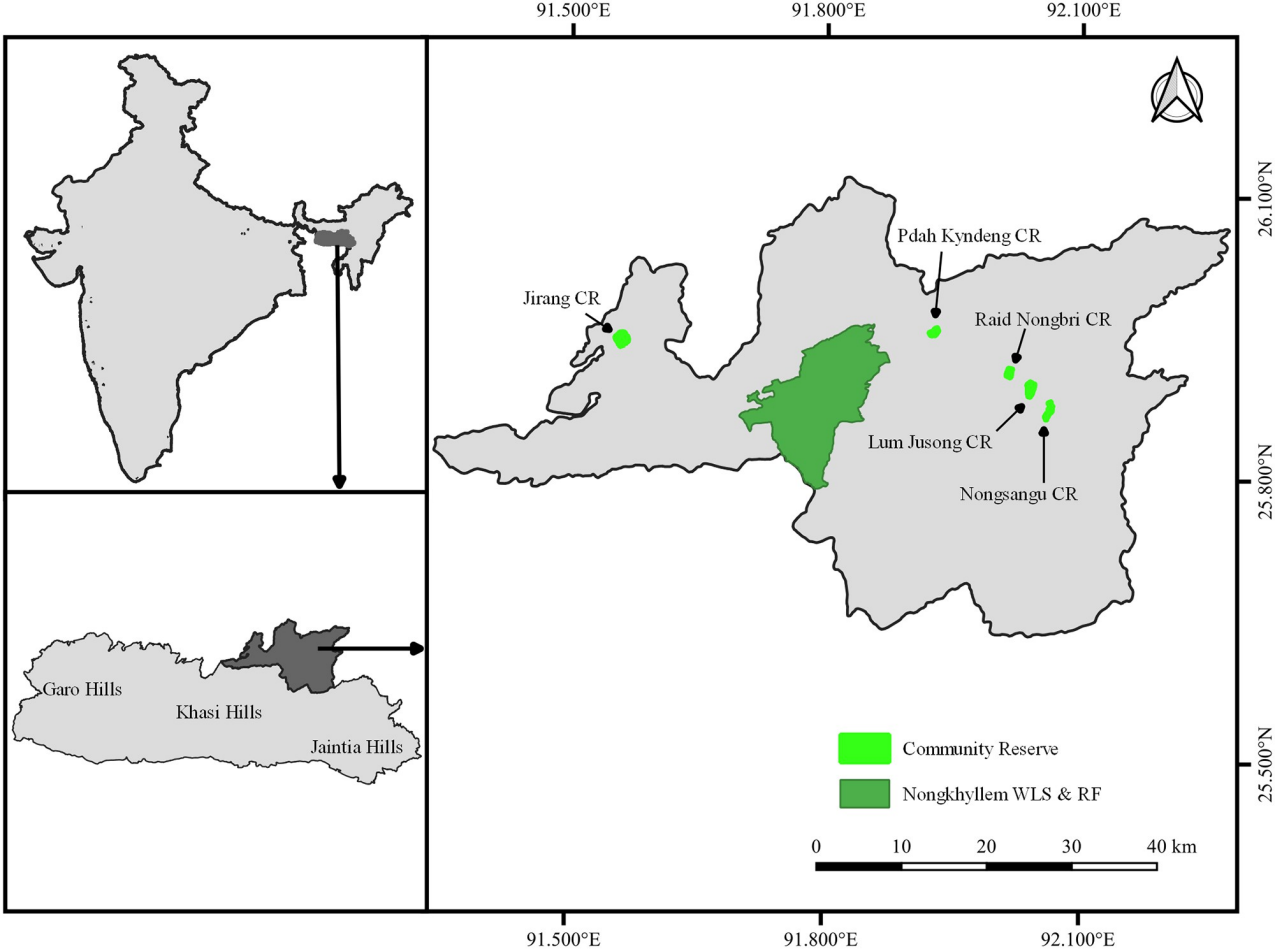
Community reserves surveyed in Ri Bhoi district of Meghalaya. Republished from GADM database (https://gadm.org/) under a CC BY license, with permission from Global Administrative Areas (GADM), original copyright 2012.

There are five CRs in Ri Bhoi district of Khasi Hills, Meghalaya (Fig 1), *viz*. Jirang CR (JCR), Nongsangu CR (NCR), Lum Jusong CR (LJCR), Pdah Kyndeng CR (PKCR) and Raid Nongbri CR (RNCR) (Table 1). We conducted the study between November 2018 and August 2019. All these reserves lie between 340–950 m above sea level. The forests in these CRs are mostly secondary in nature with the villagers having undertaken assisted natural regeneration of the forests with the help of the state forest department. The vegetation in these CRs is dominated by *Schima wallichi*, *Shorea robusta*, *Holarrhena sps*., *Castanopsis indica*, *Ficus hispida*, *Dendrocalamus hamiltonii* and *Bambusa tulda*. The areas adjacent to these CRs are under cultivation of *Jhum* crops, broom grass plantations and paddy fields.

**Table 1:** Sampling effort for detecting mammals and people perception about mammals in each Community Reserve of Ri Bhoi district.

Community Reserves	Major vegetation types	Area (ha)	Sampling effort				villages and number of villagers interviewed	
			No. of trails	Day transects (km)	Night transects (km)	No. of camera traps (Camera trap days)	Village	n
JCR	EF, MSEF, MDF, SF, BB, SL	204	14	28.16	6.89	18 (370)	Mynnar, Nongsier and Nongthymmai (5 in each village)	15
NCR	MSEF, PF, BB, SL, CA	100	9	28.45	6.38	14 (178)	Nongsangu	15
LJCR	MDF, BB, SL	130	7	28.73	10.20	14 (224)		
RNCR	MDF, BB, SL	70	7	13.43	7.04	8 (115)	Kbet Nongbri, Umsaw Nongbri	30
PKCR	MDF, BB, SL	75	7	13.03	5.82	6 (132)	Pahamshken	15
All CRs		579	44	111.80	36.33	60 (1019)	All villages	75

JCR = Jirang CR; NCR = Nongsangu CR; LJCR = Lum Jusong CR; RNCR = Raid Nongbri CR; PKCR = Pdah Kyndeng CR

EF = Evergreen Forest; MSEF = Moist Semi-evergreen Forest, MDF = Moist Deciduous Forest, SF = Sal dominated Forest, BB = Bamboo brake, SL = Scrub land, PF = Pine Forest, CA = Cultivation area

A Community Reserve Management Committee (CRMC) comprising of four members of the local community and one representative from the forest department manages each of these CRs. JCR is owned by Hima Jirang, which is the highest traditional administrative institution in Jirang territory and was notified as a CR in 2014. NCR was notified in 2014 and comes under the ownership of the Nongsangu village. LJCR, which is a sacred grove, was notified in 2016 and comes under the ownership of the local church of Nongsangu village. PKCR was notified in 2014 and comes under the ownership of Raid Nonglyngdoh (Nonglyngdoh territory), a traditional administrative institution managing a smaller territorial unit consisting of a few villages. RNCR was notified in 2014 and comes under the ownership of Raid Nongbri (Nongbri Territory). This CR is a sacred grove, although customary rituals had not been practiced for decades. The vegetation types found in the study area includes 3C/C3b East Himalayan Moist Mixed Deciduous Forest, 3C/C1 Khasi Hill Sal, 2/2S1 Secondary Moist Bamboo Brakes and 8B/C2 Khasi Sub-Tropical Wet Hill Forest [30].

### Data collection

#### Recce survey

Considering the difficult terrain, reconnaissance or recce method [31] was used for sampling mammal species. Pre-existing trails (44 nos.) that were passable were identified and marked. These trails were selected such that the entire CR was covered and were representative of all vegetation types. Two observers walked slowly in the morning (6 am to 9 am) for three-seven consecutive days for detecting both direct and indirect signs (scat, pellets or droppings, pug marks, hoof marks and calls) of mammal species. We recorded geo-coordinates, time, date and number of individuals sighted of all species encountered. We also recorded the perch height and tree height of arboreal species. We also conducted nocturnal surveys between 7 pm and 9 pm. During the survey, we flashed the light to detect the species for the eye reflection. Once the animal was spotted, the animal was carefully approached and the species was observed using a binocular and identified. The trail length was recorded using the track log in the handheld global position system (Garmin e Trex60).

#### Camera trapping

**Fig 2:**
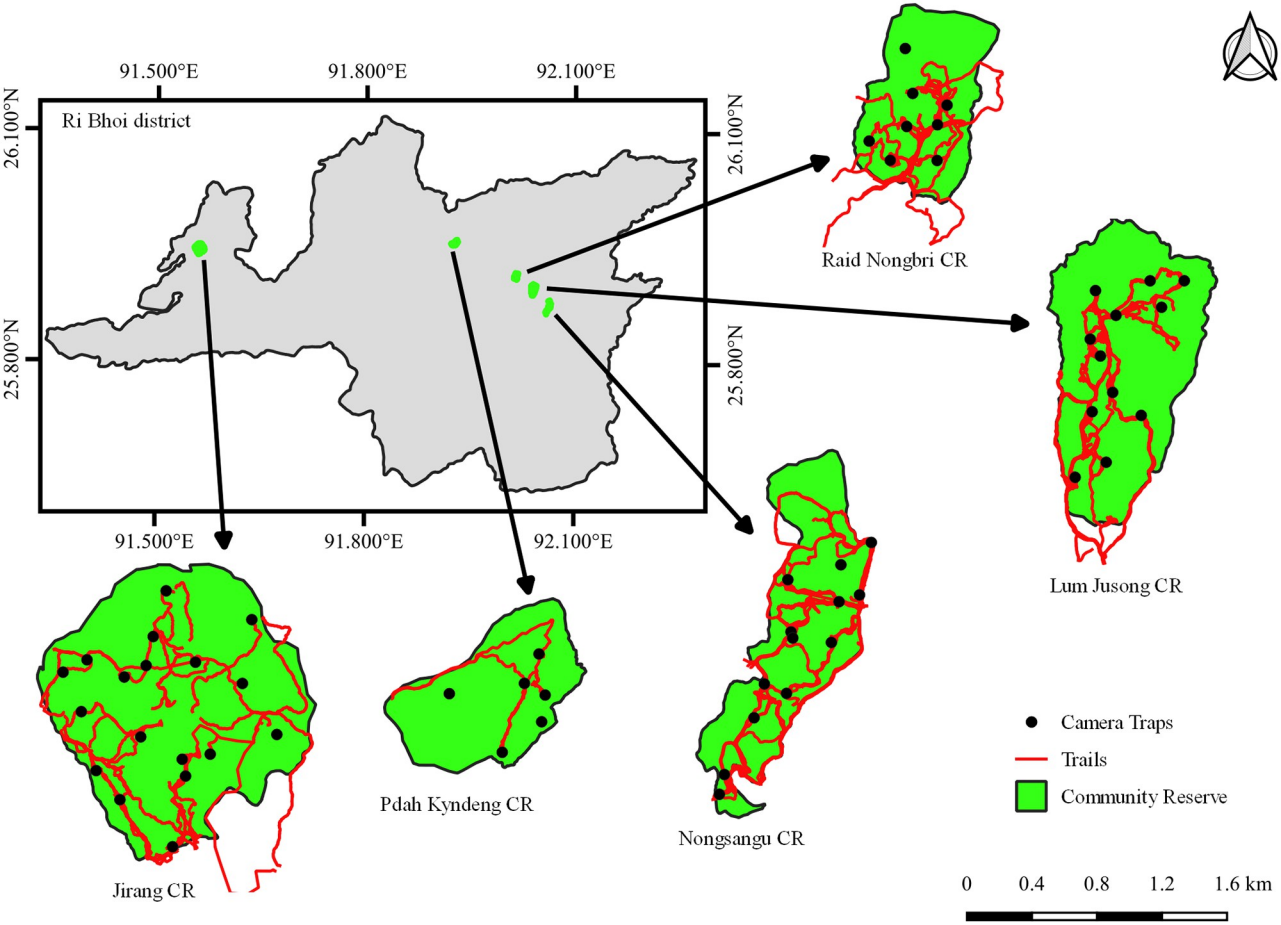
Map showing the camera trap locations and survey trails (both diurnal and nocturnal) for detection of mammals in each CRs of Ri Bhoi district. Republished from GADM database (https://gadm.org/) under a CC BY license, with permission from Global Administrative Areas (GADM), original copyright 2012.

Based on the recce survey, a total of 60 camera trap stations (Cuddeback IR Model) were placed in the CRs (Table 1, Fig 2). Since all the CRs are lesser than 200 ha, each CR was divided into 10-ha grids and a single passive infrared camera was placed within each of the grids, at a minimum distance of 100 m between each camera trap station. To maximize the probability of capturing a wide array of mammal species across different taxa, the cameras were placed non-randomly along animal trails, existing human trails, crossing points on streams, near fruiting trees or at locations where indirect signs of wildlife presence were recorded [32]. To improve the detection of a range of mammals of various sizes, the cameras were placed 30–40 cm above the ground [32] and the mode was set to take 3 burst images followed by a 10s video whenever motion was detected. Camera traps were deployed for 1019 camera trap days that varied from 115 to 370 camera trap days between the CRs. Geo-coordinates, altitude and habitat characteristics were recorded for each camera trap station.

#### People’s perception and hunting practices

Informal interviews were first conducted with CRMC members to discuss about the historical and cultural context of the CRs as well as briefing them about the study. Using cluster sampling villages surrounding each CR were selected and 5–15 villagers were randomly selected from each village and interviewed using semi-structured questionnaires with the assistance of local field guides [33]. All the interviews were conducted in Khasi, or translated into the local dialect as and when required by the local guides, and then later transcribed into English. A total of 75 villagers (63 males and 12 females) from 7 villages were interviewed (Table 1). Before undertaking the survey in each village, the village headmen were first apprised of the study and permission was acquired from them to conduct the survey in their village. Verbal informed consent was secured from each survey participant prior to the interview in accordance with guidelines from the British Sociological Association Statement of Ethical Practice [34]. The participants were further intimated that they had the right to refuse participation in the survey, could end the interview at any time and could refuse to respond to any question. Written consent was not acquired due to the low literacy levels of the participants as well as their apprehension in signing any document. Since the questionnaire survey was undertaken in 2020 during the Covid-19 pandemic, safety protocols as issued by WHO were strictly adhered to.

Local names of mammals were acquired from the local guides and these were used during the interview in addition to the mammal field guide books [35, 36] as reference for the participants. Photographs of mammals found in Meghalaya were shown to the participants and they were asked to identify the species that they had encountered. They were then asked about their perceptions on the population status of each species they had encountered, existing taboos and beliefs, wildlife conflict, bushmeat consumption, and hunting practices in their village (S1 File).

### Analysis

Species accumulation curves (SAC) were generated for all the CRs separately as well as for the pooled sample data using the data acquired from the day-time survey, night-time survey and camera trapping. Then, the species richness was estimated through extrapolation. All analysis was carried out using the ‘vegan’ package in R programme [37].

#### Recce survey

Encounter rate was calculated for each diurnal and nocturnal mammal separately. Encounter rate for each species was calculated as the total number of detections divided by the total distance surveyed. Individuals/km was used as an index of relative abundance [31, 38].

#### Camera trapping

As this study is only a preliminary investigation on the presence of mammals in the CRs, only raw metrics such as relative abundance index (RAI) was calculated for each of the species captured [39]. Although this index has many drawbacks [32, 40], it is still a useful descriptor for making preliminary observations within the study area [41]. RAI was calculated as the number of animals captured per 100 trap nights. The number of camera trap days/nights was calculated from the deployment date to the retrieval date of the camera traps. Detections of individuals of the same species were considered independent if the time interval between consecutive photographs was > 0.5 hr [42]. Photos with more than one individual of the same species were considered as one detection for that particular species. The RAI of this study was compared to RAI values of other studies that were conducted in similar habitats of South and Southeast Asia [43–49].

#### People’s perception and hunting practices

Exact multinomial test was carried out on the data using the ‘XNomial’ package in R programme [50, 51] to determine differences in the people’s perceptions among the five CRs. Post-Hoc analysis was carried out on the data using Binomial test. In some instances, a respondent provided multiple answers or statements to a single question. In such cases, the answers are fed into their respective category and totalled. This has led to figures not tallying to 100.

## Results

### Mammal species richness

We recorded 40 mammal species in the study area (S2 Table). As per the IUCN Red List (2020), 12 of these species are placed under various categories of threatened (1 critically endangered, 3 endangered, 8 vulnerable), 4 near threatened and 24 least concerned. The tiger (*Panthera tigris*), golden jackal (*Canis aureus*), wild dog (*Cuon alpinus*), gaur (*Bos gaurus*), wild water buffalo (*Bubales arnee*) and hog deer (*Axis porcinus*) were reported by local people to occur before but had now become extinct in the study area.

### Relative abundance

The SAC did not reach asymptote for all the CRs for both camera trapping and recce survey (S2 File). This indicates that our sampling efforts were not sufficient to detect all the species present in the CRs. When all the data points of the camera trap survey were pooled together, the SAC reached asymptote. However, this was not the case for the recce survey (both day-time and night-time surveys).

**Fig 3:**
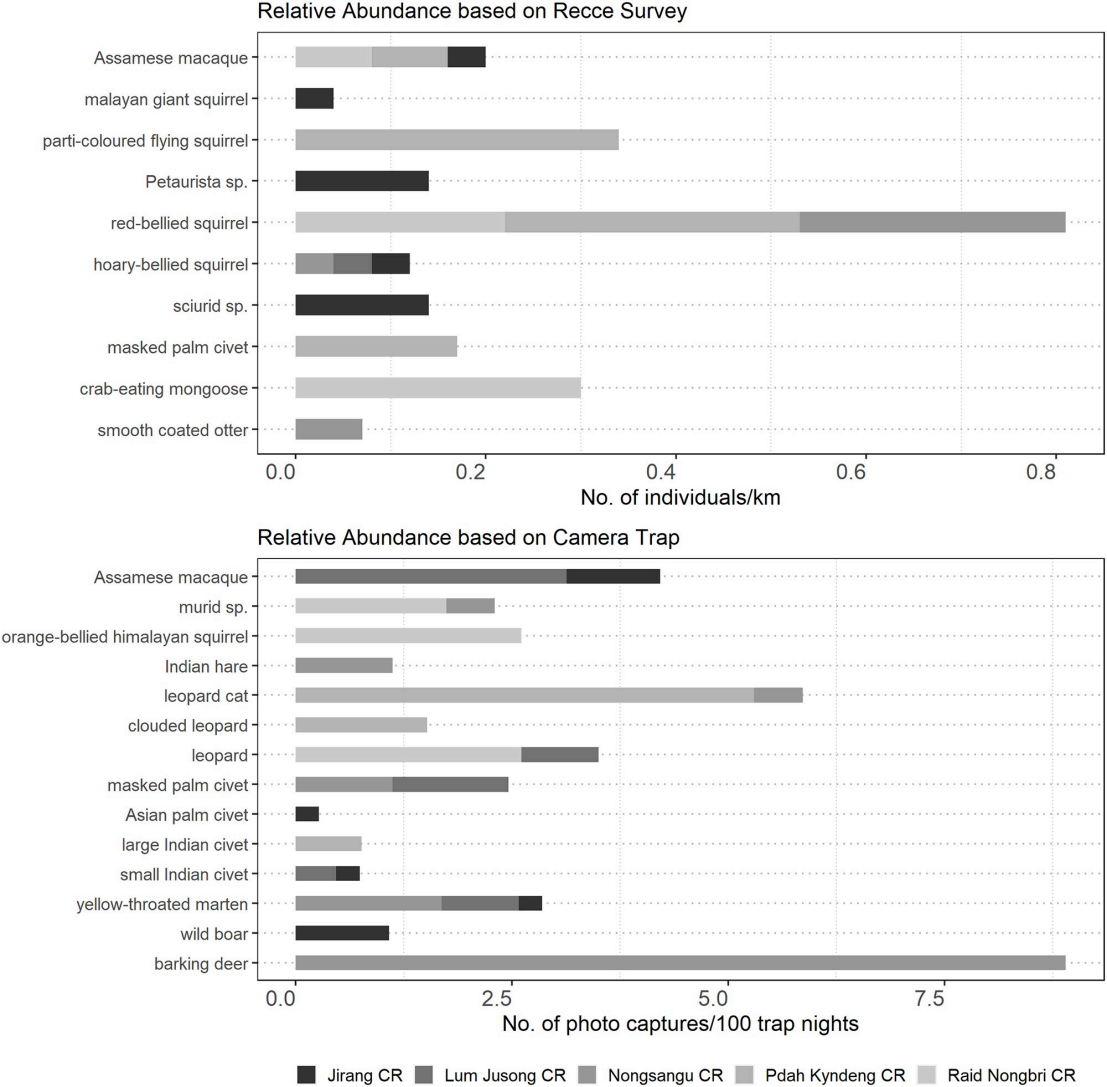
Relative abundance of mammals (no. of individuals/km) during both day-time and night-time surveys as well as for camera trapping (CR = animals/100 trap night) of all species in the Community Reserves of Ri Bhoi district.

We detected 12 and 4 mammal species through day-time and night-time survey respectively (Fig 3). However, the extrapolated species richness was estimated to range between 15.05–42.77 and 5.18–8.60 species for day-time and night-time survey respectively (S2 File). Among all the detected species, the red-bellied squirrel (*Calloscuirus erythraeus*) had the highest encounter rate while other small to medium sized mammals rarely being sighted. Species such as elephant (*Elephas maximus*), sambar (*Rusa unicolor*), barking deer (*Muntiacus vaginalis*), Chinese pangolin (*Manis pentadactyla*), hoolock gibbon (*Hoolock hoolock*) and capped langur (*Trachypithecus pileatus*) were detected only through indirect signs.

We photo-captured 14 species in all the CRs (Fig 3). However, the extrapolated species richness was estimated to range between 15.48–17 species. The capture rate of barking deer was highest, followed by Assamese macaque (*Macaca assamensis*), leopard (*Panthera pardus*) and leopard cat (*Prionailurus bengalensis*). The yellow-throated marten (*Martes flavigula*) was photo-captured in three of the CRs while other species such as barking deer, wild boar (*Sus scrofa*), Asian palm civet (*Paradoxurus hermaphroditus*), large Indian civet (*Viverra zibetha*) and clouded leopard (*Neofelis nebulosa*) were captured in only one of the CRs.

### Respondent characteristics

Survey respondents were largely men (84.0%) and primarily engaged in farming (76.0%). Respondents were aged between 30–50 years (42.7%), >50 years (41.3%) and <30 years (14.7%, S1 Table).

### Hunting practices

Villagers hunt opportunistically (33.3%), which is followed by more hunting in winter (25.3%), while less during the dry season (9.3%, Table 2). A wide range of mammals are primarily hunted for meat (45.3%), while some mammals are hunted in retaliation to crop raiding (25.3%). A few mammals are hunted for trade (8.0%) such as the Chinese pangolin which is widely hunted to meet international demands while other species such as the Bengal slow loris, wild boar, barking deer and sambar are hunted to meet local demands such as keeping as pet and for meat. Primates are usually kept as pets (2.7%) and few mammals like bear and porcupine are hunted for use in traditional medicine (2.7%). The Bengal slow loris is also hunted since local people believe seeing of loris as a bad omen or taboo (1.3%).

**Table 2:** Purpose of hunting by locals, tools used, place of hunting and season preference (n = 75).

Hunting season preference	% of responses	Species
Winter (November-February)	25.3	Macaque, crestless porcupine, squirrel, hare, Chinese pangolin, mongoose, bear, wild boar, barking deer,
Dry season (February-April)	9.3	Barking deer, sambar
Opportunistic	33.3	Elephant, Bengal slow loris, macaque, capped langur, squirrel, leopard cat, leopard, civet, mongoose, Chinese pangolin, wild boar, barking deer, sambar
No response	32.0	-
**Purpose for hunting**	**% of responses**	**Species**
Personal consumption	45.3	Bengal slow loris, macaque, capped langur, squirrel, crestless porcupine, hare, Chinese pangolin, leopard cat, civet, mongoose, wild boar, barking deer, sambar
Retaliation for crop/livestock depredation	25.3	Elephant, macaque, squirrel, crestless porcupine, leopard cat, leopard, civet, wild boar
Trade	8.0	Bengal slow loris, wild boar, barking deer, sambar, Chinese pangolin
To keep as pet	2.7	Bengal slow loris, macaque, squirrel, hare
Traditional medicine	2.7	Crestless porcupine, bear
Taboo	1.3	Bengal slow loris
No response	14.7	-
**Tool used**	**% of responses**	**Species**
Snare/Traps/catapults	28.0	Bengal slow loris, macaque, capped langur, squirrel, crestless porcupine, hare, Chinese pangolin, leopard cat, leopard, civet, mongoose
Rifle	9.3	Elephant, leopard cat, leopard, bear, wild boar, barking deer, sambar
Other	6.7	Bengal slow loris, macaque, crestless porcupine, squirrel, Chinese pangolin
No response	56.0	-
**Place of hunting**	**% of responses**	**Species**
Jhum Field	29.3	Macaque, capped langur, crestless porcupine, hare, Chinese pangolin, civet, wild boar, barking deer
Village Forest	28.0	Elephant, Bengal slow loris, crestless porcupine, Chinese pangolin, leopard cat, civet, mongoose, bear, wild boar, barking deer, sambar
Home Garden/Village	9.3	Bengal slow loris, squirrel, crestless porcupine, leopard cat, leopard
No response	33.3	-

Different tools or methods are used for hunting of various species (Table 2). Although 9.3% of them hunted using rifles, majority of the hunters use snares or traps (28%) to capture the mammals. Rifles are primarily used for ungulates and large carnivores. Other methods that hunters use include digging burrows (2.7%) for fossorial mammals such as Chinese pangolin, bamboo rat, porcupines and rodents; catapults (1.3%) for small arboreal mammals like squirrels; and by hand (1.3%) for slow moving mammals like the Bengal slow loris. Most of them hunted in their *Jhum* fields (29.3%), nearby community forests (28%), occasionally within their home gardens or village (9.3%).

### Wild meat preference and non-preference

**Fig 4:**
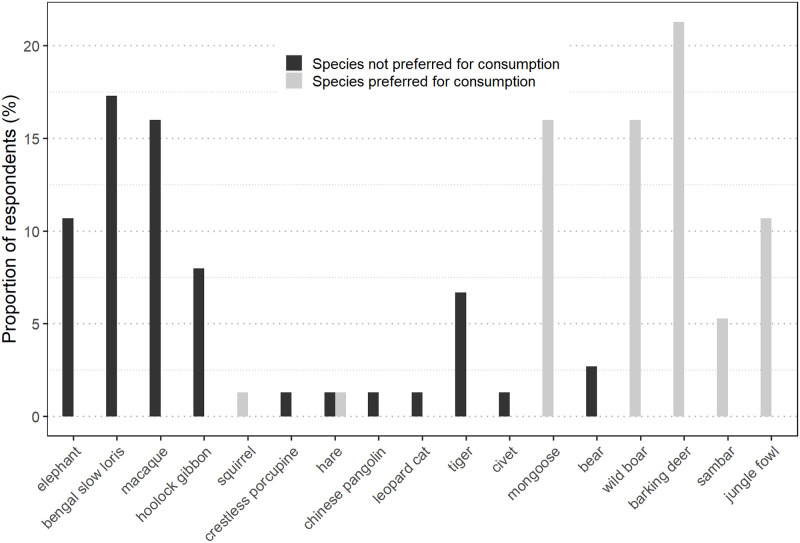
Species wise meat preferences of local community (n = 75) in five CRs of Ri Bhoi district.

Barking deer (21.3%), wild boar (16%) and mongoose (16%) are the most preferred mammals for local consumption (Fig 4). Sambar (5.3%) is also a preferred species in areas where it is still exists, while species such as primates (41%), elephants (10.7%) and carnivores (12%) apart from mongoose are not preferred. 56% of respondents did not indicate their wild meat preferences.

### Perception on wildlife population status and trends

#### Population trend

**Fig 5:**
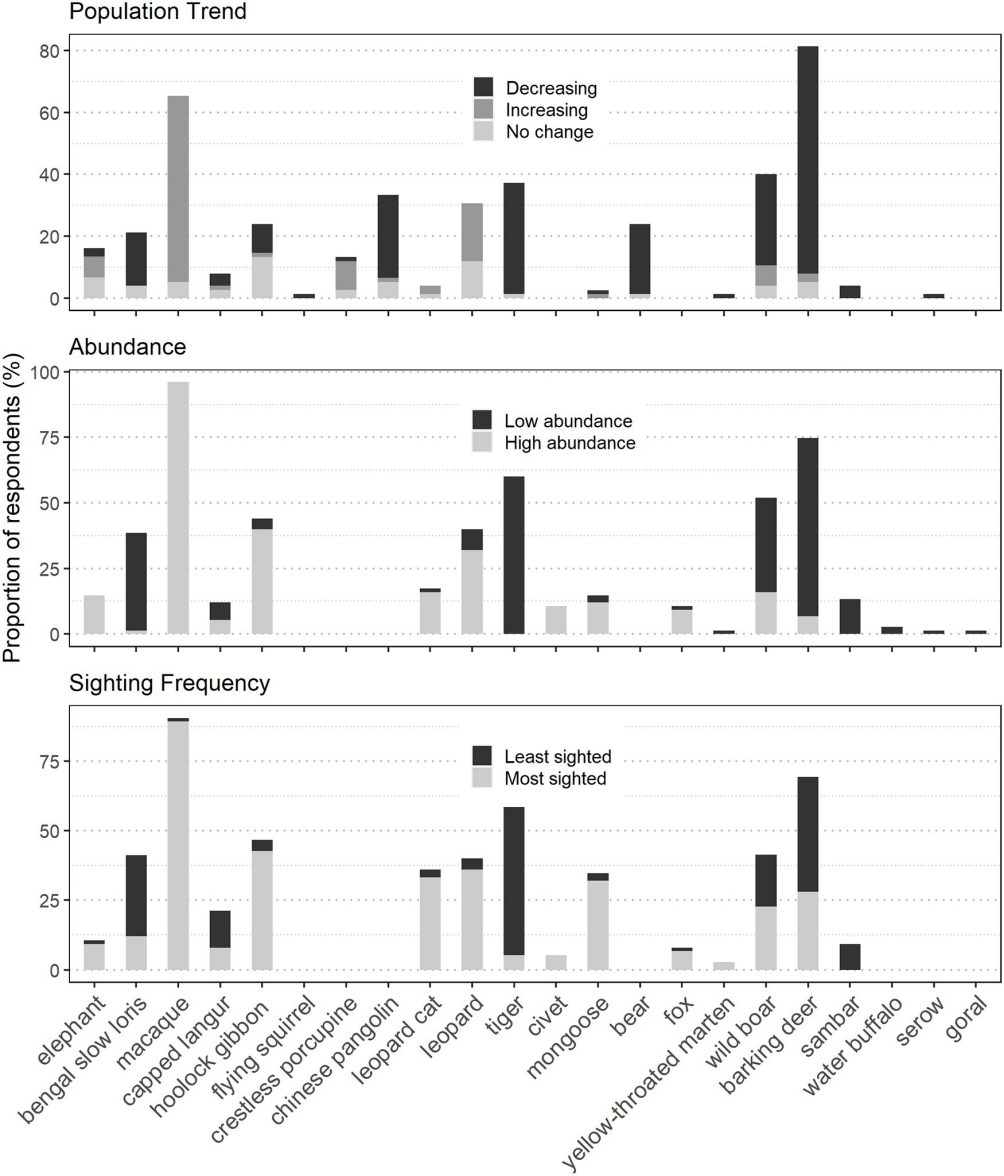
Respondents’ perceptions on population trend, species abundance and their frequency of sighting species in select CRs of Ri Bhoi district.

Respondent perceptions varied depending on the species and CR (Fig 5 and S3 Table). Among primates, Assamese macaques were reported to have increased in number (60.0%) in four CRs except in PKCR while 5.3% reported its population stable. The remaining 34.7% respondents did not comment on the macaque’s population trend. Populations of Bengal slow loris were reported to have declined (17.3%) only by respondents of RNCR. 4% reported it is stable while the remaining 78.7% respondents did not comment on the loris’ population trend. Hoolock gibbon was reported to have decreased in number (9.3%). Majority of these respondents were from LJ/NCR (Lum Jusong CR and Nongsangu CR are managed by members of the same village, hence they have been clubbed together and will be represented as LJ/NCR). 13.3% reported its population is stable while 1.3% reported increased in population. The remaining respondents (76.1%) did not comment on the hoolock gibbon’s population trend.

Among ungulates, barking deer was reported to have declined in numbers by most respondents (73.3%) in all CRs. Only 5.3% reported it is stable and 2.7% reported that its population has increased in JCR. Population of wild boar was reported to have declined (29.3%). Most of the respondents who reported this trend were from PKCR. Only 4% reported it is stable and 6.7% reported that it has increased in JCR. The remaining 60% did not comment on its population.

Perceptions on population trends of elephants were only reported by respondents in JCR. 13.4% had reported either an increase or a stable population of elephants. Only 2.7% reported that it has decreased. 84.9% of the respondents did not comment on its population.

Among carnivores, tiger (36%) and bear (22.7%) were reported to have decreased in population. Leopard population was reported only by respondents from RNCR to have increased (18.7%) or remained stable (12%). The remaining 69.3% did not comment on its population. Chinese pangolin was reported to have declined in numbers (26.7%). 5.3% reported that its population is stable and 1.3% reported that it has increased.

#### Abundance and sighting of mammals

Respondents were asked about their frequency of sighting different mammal species as well as their perceptions on their current abundance (Fig 4 and S3 Table). Among primates, macaque was the most observed (89.3%) and abundant mammal (96.0%). Hoolock gibbon was sighted by almost half of the respondents (42.7%) and is believed to be in abundance (40.0%). Only a few respondents (4.0%) from LJ/NCR are of the opinion that the species has become less abundant compared to the past. The Bengal slow loris is believed to have become rare by more than a third of the respondents (37.3%).

Among ungulates, barking deer was rarely sighted by almost half of the respondents (41.3%) and majority (68.0%) believe it has become one of the least abundant species. Wild boar and elephant were seen often mostly by JCR respondents and they were also the only ones to report on the abundance of barking deer (5.3%), along with wild boar (16.0%) and elephant (14.7%). Respondents from other CRs, however, believe that they have become rare. JCR respondents were the only ones to report rare sighting of sambar (9.3%) while in other CRs, respondents did not mention seeing sambar. Respondents from all CRs except RNCR reported on sambar becoming rare as well.

Among carnivores, more than a third of the respondents have seen leopard (36.0%) and leopard cat (33.3%), with majority of the respondents being from RNCR. RNCR respondents also believe that the leopard is still abundant while about half the LJ/NCR respondents believe that leopard cat is still abundant. About one-third of the respondents also reported on observing mongoose often, with majority of PKCR respondents (16.0%) reporting it. PKCR respondents (8.0%) also believe that mongoose and civet are still abundant. About half of the respondents reported on rarely sighting tiger and about two-thirds are of the perception that tiger has become rare. About a quarter of respondents reported on rarely sighting bear and it being very rare.

About a quarter of the respondents reported Chinese pangolin to being among the least sighted mammals and close to half of the respondents stated that it has become rare.

#### Loss of wildlife

Majority of the respondents stated that habitat loss and degradation are the main causes that had led to the extirpation of wildlife in their area whereas only a few of the respondents attributed it to historical hunting as well (Table 3). All PKCR respondents (100%) and close to half of the JCR respondents (46.7%) stated that the main reason that led to the extirpation of wildlife is habitat loss while only a few LJ/NCR and RNCR respondents (13.3% and 3.3% respectively, Table 3) cited the same reason. Most respondents from RNCR (90.0%) and LJ/NCR (80.0%) blamed degradation of forests catalysed by logging. LJ/NCR respondents (66.7%) also attributed it to *Jhum* cultivation and forest fires to causing loss of wildlife. A few respondents from JCR (13.3% and 6.7%) and RNCR (6.7%) also blamed wildlife hunting or retaliatory killings to causing loss of wildlife whereas majority did not think that hunting was a major cause for loss in wildlife.

**Table 3:** Locals (n = 75) views on wildlife and hunting prevalence.

Statements	PKCR* (n = 15)	LJ/NCR* (n = 15)	RNCR* (n = 30)	JCR* (n = 15)	Log-likelihood	P
**Cause for loss of wildlife** ^+^						
There are no more dense forests or large forest tracts as compared to the past where wild animals can stay	100.0	13.3	3.3	46.7	-14.7	**0.00**
Forests have degraded due to indiscriminate felling of trees	6.7	80.0	90.0	0.0	-16.9	**0.00**
Burning of forests and Jhum cultivation have led to degradation of forests and wildlife	0.0	66.7	0.0	0.0	-16.1	**0.00**
Historical hunting and poaching had led to the decline of wildlife in the area	0.0	0.0	6.7	13.3	-2.3	0.31
Killing of wild animals in retaliation to crop raiding have caused their decline in population	0.0	0.0	0.0	6.7	-1.6	0.60
**Hunting prevalence**						
Hunting has completely stopped	60.0	0.0	0.0	6.7	-12.8	**0.00**
Hunting has decreased over the years	40.0	40.0	0.0	93.3	-15.6	**0.00**
Hunting has increased over the years	0.0	0.0	0.0	0.0	-	-
**Cause of decline in hunting** ^+^						
Hunting has reduced or stopped due to low abundance of wildlife compared to the past	93.3	0.0	0.0	0.0	-22.5	**0.00**
Hunting has reduced or stopped because forest cover has become very less	6.7	0.0	0.0	6.7	-1.8	0.52
Hunting has reduced or stopped because people have become more educated and aware about the wildlife act of the government	0.0	0.0	0.0	26.7	-6.4	**0.00**
Hunting has reduced or stopped because of the efforts of the state wildlife dept. to create awareness about the WPA, 1972	0.0	0.0	0.0	73.3	-17.7	**0.00**
**Crop depredation**						
Elephant	0.0	0.0	0.0	80.0	-19.3	**0.00**
Macaque	93.3	100.0	0.0	93.3	-22.0	**0.00**
Squirrel	0.0	20.0	3.3	0.0	-3.5	0.14
brush-tailed porcupine	0.0	6.7	0.0	0.0	-1.6	0.60
crestless porcupine	26.7	46.7	13.3	33.3	-2.3	0.24
wild boar	80.0	0.0	10.0	93.3	-17.0	**0.00**
Hare	0.0	0.0	0.0	66.7	-16.1	**0.00**
Leopard	0.0	0.0	33.3	0.0	-9.2	**0.00**
Tiger	0.0	0.0	0.0	20.0	-4.8	**0.00**
mongoose	0.0	0.0	6.7	0.0	-1.8	0.52
Bear	6.7	0.0	0.0	0.0	-1.6	0.60

*percentages may not tally to 100 as a single respondent may have provided multiple statements and some respondents had not provided any answer/statement.

^+^No respondent provided any answer that is in disagreement with the above statements.

#### Hunting prevalence

Most respondents from PKCR (100%), LJ/NCR (40%) and JCR (100%) stated that hunting has stopped or reduced in their respective villages (Table 3). Almost all respondents from PKCR (93.3%) stated that this was primarily driven by low abundance of wildlife, whereas most respondents from JCR cited habitat loss (73.3%) and awareness of the community (26.7%). RNCR respondents did not provide any statement regarding hunting prevalence.

#### Human-wildlife conflict

In RNCR (where leopard was also photo-captured) about a third of respondents (33.3%) stated that leopard causes livestock damage, while in JCR few respondents (20%) blamed tiger, although tiger was not found in the area by the authors (Table 3). Respondents from other CRs did not mention about livestock depredation. In all CRs except RNCR, almost all respondents blamed macaque for crop damage. Respondents from PKCR (80.0%) and JCR (93.3%) also stated that wild boar causes crop damage. Only JCR respondents (80.0%) stated that elephant damages their crops. Crestless porcupine was reported by respondents from all CRs to causing crop damage.

#### Folklores and taboos

Taboos and folklores were only reported from PKCR and JCR. We report here only those taboos that have a direct impact on the species. In PKCR, since the Bengal slow loris is a rare species, people believe that it is a bad omen to see the Bengal slow loris and fear that it might cause illnesses or death to them or their family members. In JCR, villagers believe that over-exploitation of Chinese pangolin will bring bad luck to the hunter and his family. The village near LJCR and NCR, earlier had religious beliefs affiliated with the panther or the melanistic form of the leopard. They used to offer animal sacrifices to the leopard.

## Discussion

We report that the mammal abundance is relatively less in the CRs of Meghalaya but nevertheless they persist. The abundance of mammals in northeast India has been declining due to various anthropogenic pressures [20, 52, 53]. [43] reported lowest abundance of some mammals in one of the protected areas in Arunachal Pradesh, which was considered to be the lowest in the south-east Asian landscape, suggesting ‘empty forest syndrome’ in the forests of northeast India. In Meghalaya, this may also be the case as our intensive camera-trapping efforts (1 camera trap/10 ha) yielded low relative abundance of mammals, comparable to other highly hunted protected areas (S4 Table). However, unlike in other protected areas where the natural habitat may still be intact, these CRs were subjected to exploitation of natural resources (pers. comm.). One such example is Lum Jusong CR which despite it being a sacred grove was auctioned off to local timber mills prior to its notification as CR. Such historical disturbances coupled with other disturbance variables such as habitat fragmentation and isolation may have played a larger role in contributing to the low relative abundance of mammals in these CRs. Such factors are yet to be investigated and are beyond the scope of this present study.

Despite the low abundance of mammals, these CRs are still important forest patches for mammals as they are the only remnant forests in the landscape. The sighting of rare carnivores such as the elusive clouded leopard in two of the reserves, ungulates such as barking deer and wild boar despite them being preferentially hunted by villagers for consumption and large-bodied animals like elephants, sambar and bear in some of the reserves e.g., JCR which is located close to Nongkhyllem WLS and Reserved Forests of Assam, indicates their persistence in these forests. Such anecdotal records suggest the importance of these CRs for the long-term survival of these mammals in the landscape.

Despite the CRs being small (< 2 km^2^) and generally isolated, they still harbour a good number of arboreal species such as macaques, hoolock gibbon, Bengal slow loris, Malayan giant squirrel, parti-coloured flying squirrel and other sciurid species. The persistence of primates in these forests is primarily because they are not preferably hunted for consumption and are considered unappetizing as they closely resemble humans by the local indigenous community. They are, however, occasionally kept as pets. This is in contrast to other north-eastern parts of India where primates are hunted for meat, sport and indigenous rituals [54]. Other arboreal species such as parti-coloured flying squirrel probably persist because of their nocturnal nature and as such are not easily encountered by villagers.

Although responses among the different community reserves varied, the general observation was that population of several species have declined, likely driven by habitat loss and degradation. However, as has been reported for some species in Garo Hills [55], the populations of species such as macaque, wild boar, elephant, leopard and leopard cat were perceived to have increased. This may be a biased observation of the respondents as such species are more likely to be sighted by them since they depredate more on their crop and livestock. The perceived rarity of other species, however, also suggest that majority of these species have declined with respondents rarely sighting them. Similar to other parts of northeast India [10, 20, 55, 56], populations of heavily hunted species such as barking deer, wild boar, sambar and Chinese pangolin, which were earlier thought to be in abundance, have either greatly declined or have been extirpated from most parts of the study area. While signs of barking deer were observed in most of the reserves, signs of wild boar, sambar and Chinese pangolin were observed in only one or two reserves.

The frequency of hunting is likely to have declined relative to the past. But it still persists predominantly as opportunistic hunting and largely occurring more intensively during the winter and dry seasons (between November and April). This is the time when villagers harvest their crops from their *Jhum* and paddy fields as well as collect fuelwood and NTFP from community forests. February-April is also the season when farmers clear *Jhum* fields for cultivation and cattle herders burn the forests for triggering growth of new shoots for their livestock. As such they are likely to encounter more animals and thus, are more actively engaged in hunting. Villagers may prefer certain species, e.g., ungulates, over others for consumption, but they will capture and eat almost any animal they encounter. This non-selective hunting has been observed in other tropical regions as well [16]. In the recent years, although hunting has greatly reduced in the study area, some respondents attributed this pattern to a general decline in wildlife populations as well as a decrease in the forest cover. A few also attributed it to a general awareness about wildlife protection laws and efforts undertaken by the state department. An observation among other villagers who were not part of the study also suggested that the presence of reserves in the area acts as a deterrent to hunters from neighbouring villages from coming to their forests for hunting. A lack of awareness of Indian laws pertaining to wildlife probably would have led to such beliefs among villagers that hunting in community forests is not illegal [20].

Our study provides preliminary information on the importance of CRs for the persistence of mammals in a human dominated landscape which predominantly consists of agriculture fields, *Jhum* and village settlements interspersed with highly fragmented forests. These CRs may serve as refuges to remnant populations of several mammal species as well as reduce pressure from hunting akin to other protected areas of higher protection status (IUCN category II or IV). However, such roles of CRs warrant further detailed investigation, which is beyond the scope of this study.

To ensure the long term viability of these isolated remnant forests for mammals, it is essential that active restoration of degraded forests [57] and habitat connectivity of these forests is improved as in the case for strictly arboreal species such as the hoolock gibbon that spend most of its time in forest canopy [58].

CRs that are closer to conventional protected areas have also been found to provide a subsidiary support for large mammals as well [26, 27]. In these cases, large PAs serve as a “source”, especially of large mammals, to these smaller community forests that are situated near them [16]. In northeast India, where much of the land is under the control of indigenous communities, there is a great potential in developing community-based protected area networks [25]. As such, greater effort should be made towards complementing the existing government-owned protected areas by incentivizing the local community into conserving their community lands.

## Conclusion

CRs under the Wild Life (Protection) Act, 1972 serve as a useful instrument to balance between conservation priorities as well as meeting the livelihood requirements of the local indigenous communities. In northeast India, where much of the land is managed by indigenous communities [25] and where only a fraction of the land is protected (National Wildlife Database, Wildlife Institute of India, wii.gov.in/national_wildlife_database), it is difficult to expand the conventional protected area network without infringing upon the land of indigenous communities. In such a situation, CRs are a pragmatic solution as they integrate both the requirements of conservation and livelihoods of the local people. However, while increasing the coverage of CRs is essential for the long-term survival of mammals in this landscape, it is also important that mitigation measures are kept in place to reduce man-animal conflict for species such as leopards, macaques and wild boar. Measures such as predator-proofing of livestock pens, supporting villagers to grow unpalatable buffer crops, chilli fences and setting up of apiaries near forest boundaries may help deter depredation while simultaneously providing villagers with supplementary sources of income. Ex gratia payments for losses to mammals can also reduce retaliatory killings. Through our study, we have highlighted the importance of CRs for the conservation of threatened mammals in a highly fragmented landscape in Meghalaya.

## Supporting information

S1 File Questionnaire for determining people’s perception to wildlife and hunting prevalence in the Community Reserves of the Khasi Hills, Meghalaya, India. (PDF)

S2 File Extrapolated species richness of all the five CRs for each survey method derived using the ‘specpool’ function of the ‘vegan’ package in R. (PDF)

S1 Table. Characteristics of locals (n = 75) interviewed (PDF)

S2 Table Checklist of mammals found in five Community Reserves of Ri Bhoi district, Meghalaya as well as species reported by locals to no longer existing in the district. ^a-^ LC = Least Concern; NT = Near threatened; VU = Vulnerable; EN = Endangered; CR = Critically Endangered; ^b-^ Ab = Absent; Pr = Present; 1 = Camera trap; 2 = Direct sighting; 3 = Faecal deposit; 4 = Footprints/ burrow; 5 = Call; 6 = Local informant; ^c-^ J = Jirang CR; N = Nongsangu CR; L = Lum Jusong CR; R = Raid Nongbri CR; P = Pdah Kyndeng CR.
(PDF)

S3 Table Perception of respondents to trends in mammal species population, abundances and their observation of wildlife. (PDF)

S4 Table Comparison of relative abundance index (RAI—number of trap-days required to get a single photo capture of a species) derived from camera trap surveys for mammals in the Community reserves of Ri Bhoi, Meghalaya with nine other forests of south and southeast Asia. ‘-‘: Indicates not recorded; highlighted figures indicate RAI values of present study higher than the median.
(PDF)
