# Supplementary material for: Community Reserves: Their significance for the conservation of mammals in a mosaic of community-managed lands in Meghalaya, Northeast India
Source: PLoS One. 2023 Jan 26;18(1):e0280994. doi: 10.1371/journal.pone.0280994 (PMC9879402; doi:10.1371/journal.pone.0280994)
Supplement: S1 File — (PDF) [file pone.0280994.s001.pdf]

Community Reserves: their significance for conservation of mammals in a mosaic of community-managed lands in Meghalaya, Northeast India

Objective: Questionnaire for characterizing people's perception to wildlife and hunting prevalence in the Community Reserves of the Khasi Hills, Meghalaya, India

Target: Villagers

Date: Community Reserve:

Interviewer name: Interview start and end time:

Part 1: Demographics

1. Respondent information:

    Name: Sex: M/F Age:

    Village: Clan:

    Ethnicity/tribe/community: Religion:

    Occupation: Primary source of income:

    Pets kept:

Part 2: Wildlife-people relationship and perception

2. Which animals have you observed the most/least since past 5 years?

Most observed	Least observed

3. Which animals are abundant/rare in number?

Abundant	Rare

4. What is the population trend of animals?

Animal	Population trend in past 10 years (increased/decreased/remained the same)	Reason

5. Are there any local traditional stories/beliefs/taboos about any animal?

    Yes ____ No ____ Not sure ____

    5a. Which animal(s)?

    5b. Can you please describe it?

    5c. Do you believe in it? Yes ____ No ____ Not sure ____

    5d. What is your reason?

6. Have you (or anyone in your household) ever owned a wild animal?
   Yes ____ No ____ Not sure ____
   6a. Which animal (s)?
   6b. How did you get it?
   6c. When did you own it?
   6d. How long did you own it?
   6e. What happened to it?

7. Any animal that is harmful or beneficial to you? Why?

Harmful	Beneficial

8. What do you think are the main threats to wildlife?

Part 3: Hunting

9. Have you (or anyone in your household) ever gone hunting for wildlife?
   Yes ____ No ____ Not sure ____
   9a. How many times did you go hunting in the last 12 months?
   9b. How many times did you go hunting in the last 5 years?
   9c. During which time of the year do you go hunt?
10. Were you (or anyone in your household) successful in hunting?
   Yes ____ No ____ Not sure ____

What animals you hunted?	How many captured in		Weapon used?	Purpose of hunting (Commercial/Personal; medicine, food, depredation, trade, pet)	Time of year	Place of hunt (forest, jhum field etc.)
	12 months	5 years				

11. Which animal species are preferentially hunted?

12. Which animal species are not consumed?

13. How is the practice of hunting now compared to the past?
   13a. What is the reason for this?

Translated Questionnaire into Khasi language

Jingthmu: Ban sngewthuh shaphang ka jingiadei hapdeng ki briew bad ki mrad

Ka thong: Nongkyndong

Tarik: Community Reserve:

Part 1: Demographics

1. Ki jingtip jong ki nongjubab:

Kyrteng: Sex: Shynrang/Kynthei Snem:

Shnong: Kur:

Ethnicity/tribe/community: Ka niam:

Kam: Ka jingioh kamai ba kongsan:

Ki mrad/sim ba ri ha iing:

Part 2: Ka jingiadei bad jingsngewthuh ki paidbah shaphang ki mrad

2. Ki mrad aiu kiba phi shem kham bunsien ha kine ki san snem ba lah dep?

Pyndap hangne ki kyrteng jong ki mrad (e.g. u khla, ka skei, u hati, bhangsoh… kumta ter ter)

2. Ki mrad aiu kiba phi khlem da shem than eh ha kine ki san snem ba lah dep?

Pyndap hangne ki kyrteng jong ki mrad (e.g. u khla, ka skei, u hati, bhangsoh… kumta ter ter)

3. Ki mrad aiu kiba kham bun?

Pyndap hangne ki kyrteng jong ki mrad (e.g. u khla, ka skei, u hati, bhangsoh… kumta ter ter)

3. Ki mrad aiu kiba la kham duna mynta?

Pyndap hangne ki kyrteng jong ki mrad (e.g. u khla, ka skei, u hati, bhangsoh… kumta ter ter)

4. Ka jingdon jong ki jait mrad ka long kumno ha kine ki 10 snem?

Kyrteng mrad	Ka jingdon ki mrad ka kiew/hiar/ ka sah katjuh	Ka daw balei phi sngew ba ka long kumta

5. Don khanatang/jingngeit eiei shaphang ki jait mrad?
   Hooid ____ Em ____ Ym thikna re ____
   5a. Ki mrad aiu kita?
   5b. Hato batai khyndiat shaphang kata ka khanatang?
   5c. Hato phi dang ngeit shaphang kata ka khanatang?
      Hooid ____ Em ____ Ym thikna re ____
   5d. Balei?

6. Hato ma phi lane ki bahaiing jong phi ki lah ju ri mrad khlaw ne em?
   Hooid ____ Em ____ Ym thikna re ____
   6a. Ki kyrteng aiu kita ki mrad?
   6b. Phi ia ioh naei?
   6c. Mynno phi ri ia ki?
   6d. Katno snem/bnai phi ri ia ki?
   6e. Jia aiu ia ki?

7. Don kino kino ki mrad kiba iarap ne kiba ai jingmyntoi ia phi? Balei?

Pyndap hangne ki kyrteng jong ki mrad (e.g. u khla, ka skei, u hati, bhangsoh… kumta ter ter) Bad ki daw balei phi sngew kumta

7. Don kino kino ki mrad kiba wanrah ka jingsniew ia phi? Balei?

Pyndap hangne ki kyrteng jong ki mrad (e.g. u khla, ka skei, u hati, bhangsoh… kumta ter ter) Bad ki daw balei phi sngew kumta

8. Kiei tharai ki daw ba pynjulor lane kiba pynduhkait ia ki mrad hangne?

Part 3: Ka jingshikar/jingriam mrad

9. Ma phi lane ki bahaiing jong phi ki la ju ia sar ia ki mrad mynshuwa?
   Hooid ____ Em ____ Ym thikna re ____
   9a. Katno sien ha une u shi snem?
   9b. Katno sien ha kine ki san snem?
   9c. Ha ki por aiu ba kham biang ban leit sar mrad?

10. Ma phi lane ki bahaiing jong phi ki la ju ia ioh kem ne pyniap ia ki mrad mynshuwa?
   Hooid ____ Em ____ Ym thikna re ____

Mrad aiu ba phi	Katno tylli ha kine ki		Ki daw ba phi pyniap/kem mrad		Jaka ba phi shikar

pyniap ne kem?	1 snem	5 snem	Tiar aiu kiba phi pyndonkam? (e.g., suloi, ki tiar riam mrad, wait, kumta ter ter)	(ban khaii/ban pyndonkam shimet/ dawai/ ban bam/ ba ki pynjulor ia la ki jingthung/ ban khaii/ ban ri ha la iing)	Por aiom aiu	(khlaw, rep shyrti, kper, lyngkha, shnong)

11. Mrad aiu ba ki briew ki ju kham sar?

12. Mrad aiu ba ki briew kim ju kham bam?

13. Ki briew ki dang ia kham sar mynta kumba ia sar mynshuwa?
    13a. Hato batai khyndiat balei phi sngew kumta?
