# Supplementary material for: Community Reserves: Their significance for the conservation of mammals in a mosaic of community-managed lands in Meghalaya, Northeast India
Source: PLoS One. 2023 Jan 26;18(1):e0280994. doi: 10.1371/journal.pone.0280994 (PMC9879402; doi:10.1371/journal.pone.0280994)
Supplement: S2 File — (PDF) [file pone.0280994.s002.pdf]

S2 File: Extrapolated Species Richness of all the five CRs for each survey method derived using the 'specpool' function of the 'vegan' package in R.

Survey Method	Sample	No. of Species detected	extrapolated species richness				No. of sampling units
			chao	jack1	jack2	boot	
Camera trapping	Jirang CR	5	10.25	8.50	11.50	6.40	18
	Nongsangu CR	6	11.57	9.71	13.14	7.45	14
	Lum Jusong CR	5	6.86	6.86	7.78	5.86	14
	Raid Nongbri CR	3	3.00	3.00	1.07	3.30	8
	Pdah Kyndeng CR	3	3.42	3.83	3.97	3.42	6
	**All CR**	**14**	**15.48**	**16.95**	**17.00**	**15.63**	**60**
Day-time survey	Jirang CR	7	20.13	12.25	16.75	9.08	8
	Nongsangu CR	3	3.83	4.67	6.00	3.67	6
	Lum Jusong CR	4	7.38	6.25	7.42	5.01	4
	Raid Nongbri CR	3	5.25	5.25	6.75	3.95	4
	Pdah Kyndeng CR	3	4.50	4.50	5.17	3.70	4
	**All CR**	**12**	**42.77**	**19.69**	**26.19**	**15.05**	**26**
Night-time survey	Jirang CR	2	2.75	3.50	4.50	2.63	4
	Nongsangu CR	0	0.00	0.00	0.00	0.00	2
	Lum Jusong CR	0	0.00	0.00	0.00	0.00	3
	Raid Nongbri CR	0	0.00	0.00	0.00	0.00	3
	Pdah Kyndeng CR	2	2.33	2.67	2.83	2.33	3
	**All CR**	**4**	**8.20**	**6.80**	**8.60**	**5.18**	**15**

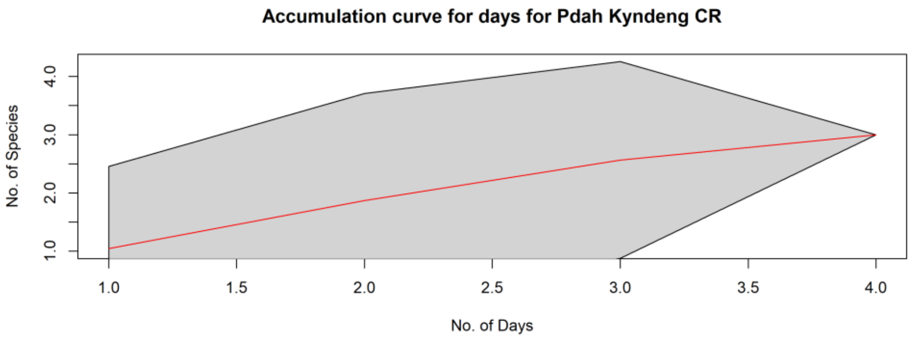

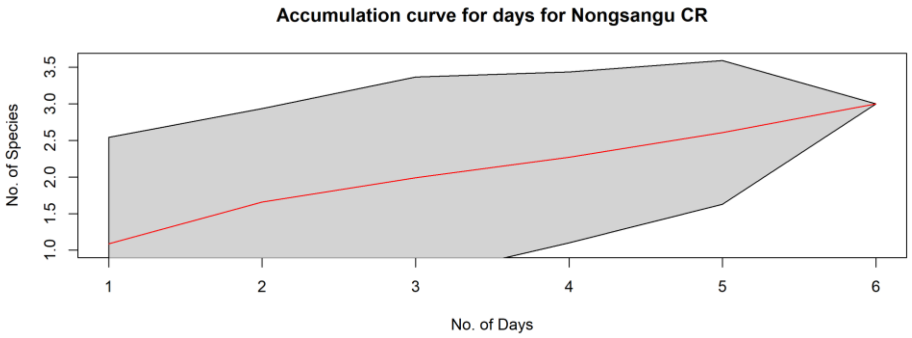

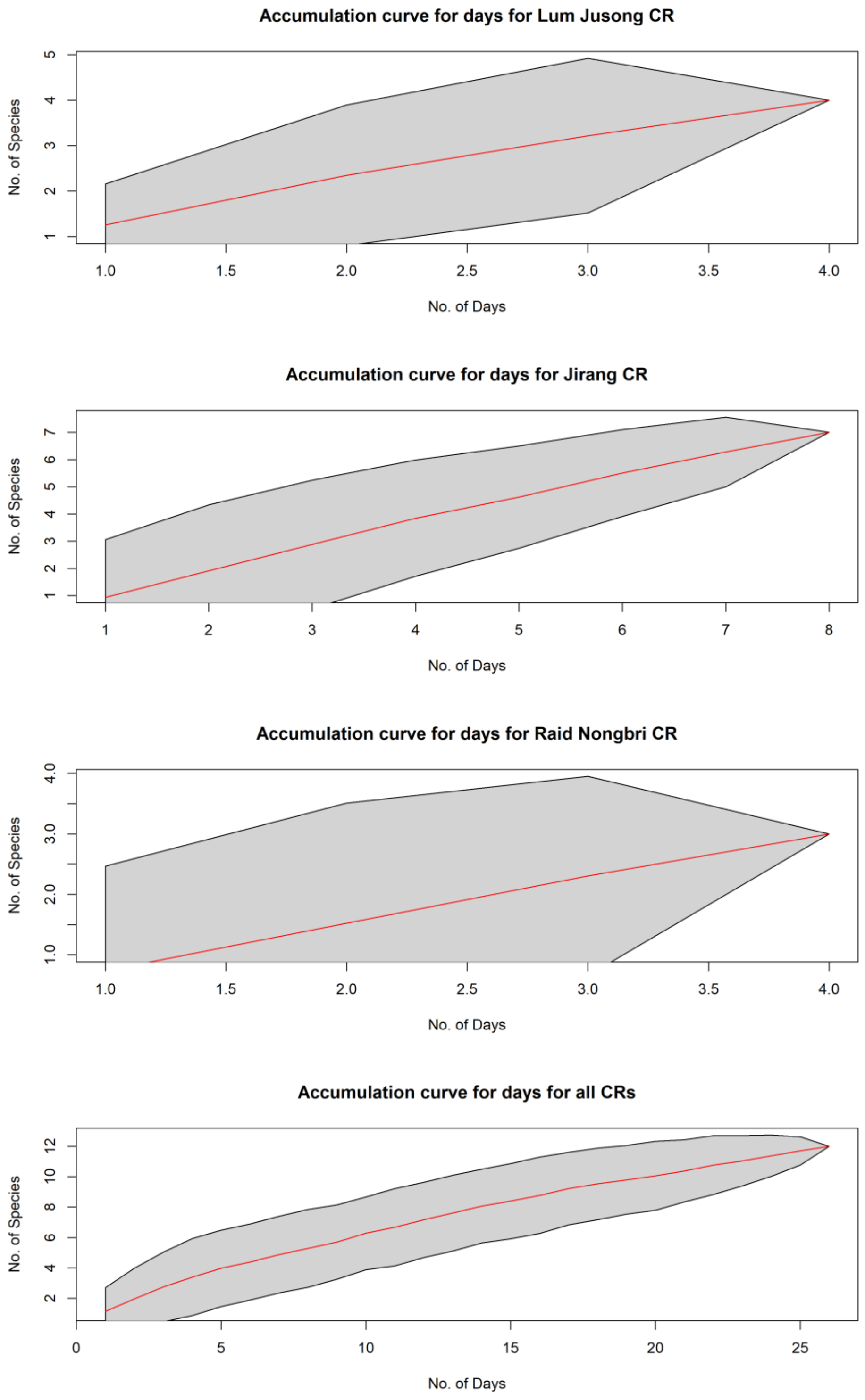

Fig. Species accumulation curve for day-time survey of all CRs

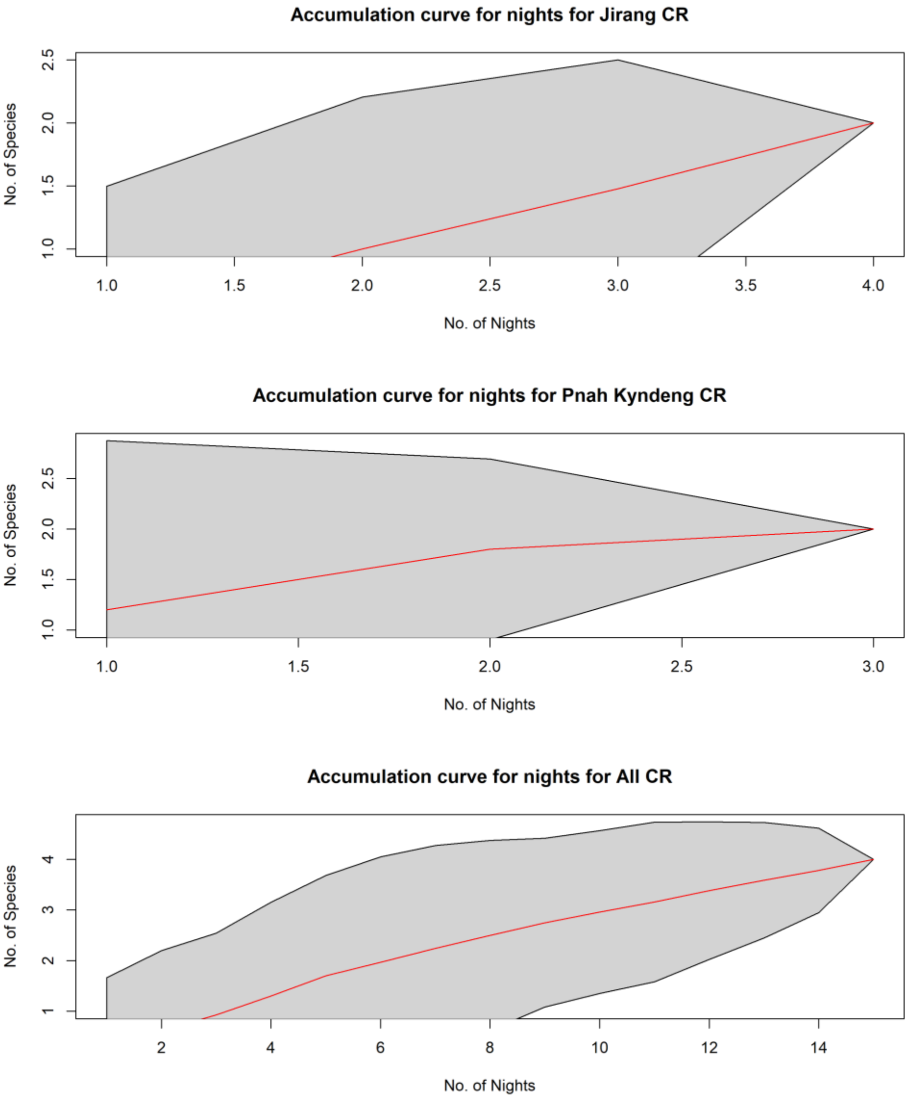

Fig. Species accumulation curve for night-time survey of all CRs

Accumulation curve for CT for Pdah Kyndeng CR

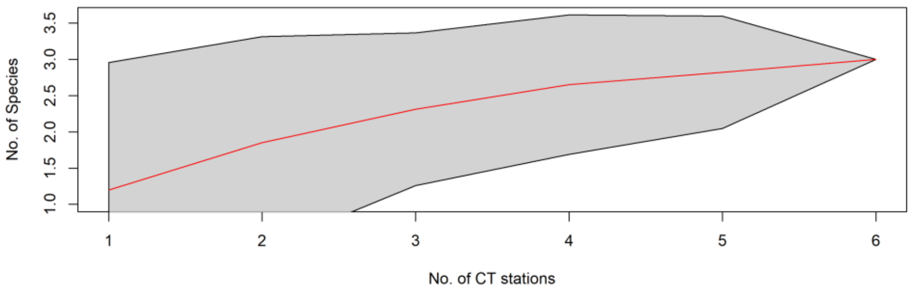

Accumulation curve for CT for Raid Nongbri CR

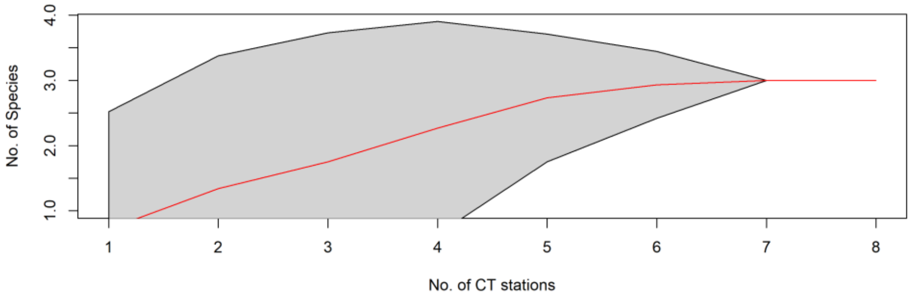

Accumulation curve for CT for Nongsangu CR

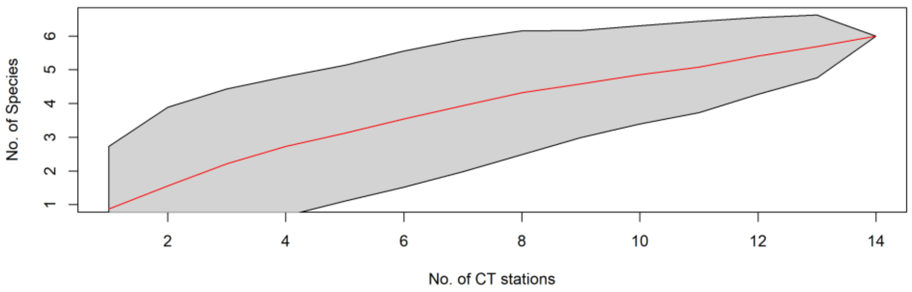

Accumulation curve for CT for Lum Jusong CR

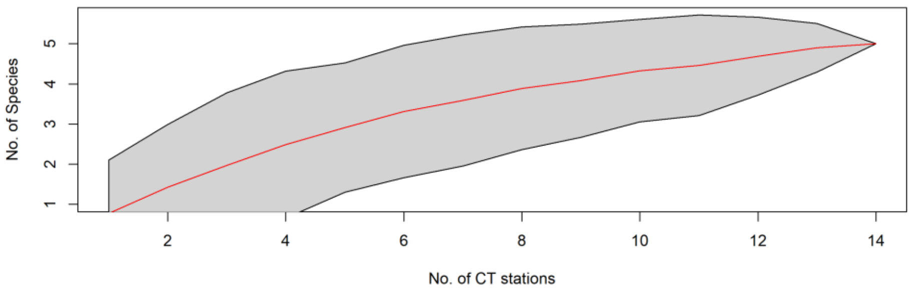

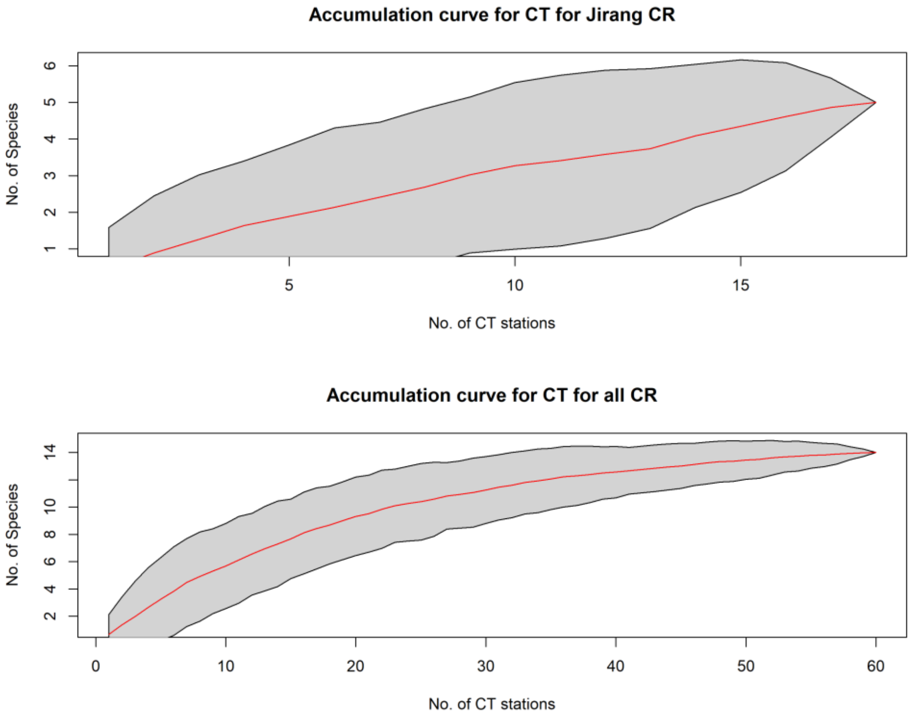

Fig. Species accumulation curve for camera trap survey of all CRs
