# Supplementary material for: Community Reserves: Their significance for the conservation of mammals in a mosaic of community-managed lands in Meghalaya, Northeast India
Source: PLoS One. 2023 Jan 26;18(1):e0280994. doi: 10.1371/journal.pone.0280994 (PMC9879402; doi:10.1371/journal.pone.0280994)
Supplement: S1 Table. Characteristics of locals (n = 75) interviewed — (PDF) [file pone.0280994.s003.pdf]

Community Reserves: their significance for conservation of mammals in a mosaic of community-managed lands in Meghalaya, Northeast India

S1 Table. Characteristics of locals (n=75) interviewed

**No. of villagers interviewed: 75**								
**Age**	**%**	**Ethnicity**	**%**	**Sex**	**%**	**Occupation**	**%**	**Religion**
<30	14.7	Bhoi	80.0	Male	84.0	Farmer	76.0	Christianity
30-50	42.7	Mnar	17.3	Female	16.0	Other	24.0	
>50	41.3	Rabha	2.7					
