# Supplementary material for: Community Reserves: Their significance for the conservation of mammals in a mosaic of community-managed lands in Meghalaya, Northeast India
Source: PLoS One. 2023 Jan 26;18(1):e0280994. doi: 10.1371/journal.pone.0280994 (PMC9879402; doi:10.1371/journal.pone.0280994)
Supplement: S2 Table — a- LC = Least Concern; NT = Near threatened; VU = Vulnerable; EN = Endangered; CR = Critically Endangered; b- Ab = Absent; Pr = Present; 1 = Camera trap; 2 = Direct sighting; 3 = Faecal deposit; 4 = Footprints/ burrow; 5 = Call; 6 = Local informant; c- J = Jirang CR; N = Nongsangu CR; L = Lum Jusong CR; R = Raid Nongbri CR; P = Pdah Kyndeng CR. (PDF) [file pone.0280994.s004.pdf]

Community Reserves: their significance for conservation of mammals in a mosaic of community-managed lands in Meghalaya, Northeast India

Table S2. Checklist of mammals found in five Community Reserves of Ri Bhoi district, Meghalaya as well as species reported by locals to no longer existing in the district.

Sl. No.	Order / Scientific name	Common name	IUCN Status[a]	Wildlife Protection Act, 1972 Schedule/ Status	Present Status[b]	Year last observed by locals	CR[c]
	Proboscidea						
1	*Elephas maximus*	Asian elephant	EN	Sch I (Part I)	Pr (4,6)	2019	J
	Primates						
2	*Nycticebus bengalensis*	Bengal slow loris	EN	Sch I (Part I)	Pr (6)	2014	J,N,L,R,P
3	*Macaca assamensis*	Assamese macaque	NT	Sch II (Part I)	Pr (1,2,5,6)	2018	J,N,L,R,P

4	*Macaca mulatta*	rhesus macaque	LC	Sch II (Part I)	Pr (6)	-	J,N,L,P
5	*Trachypithecus pileatus*	capped langur	VU	Sch I (Part I)	Pr (2,5,6)	-	J,N,L,R,P
6	*Hoolock hoolock*	hoolock gibbon	EN	Sch I (Part I)	Pr (2,5,6)	2019	J,N,L,R,P
	Rodentia						
7	*murid sp.*		LC	Sch V	Pr (1,6)	-	J,N,L,R,P
8	*Ratufa bicolor*	malayan giant squirrel	NT	Sch II (Part I)	Pr (2,6)	-	J,P
9	*Hylopetes alboniger*	parti-coloured flying squirrel	LC	Sch II (Part I)	Pr (1)	-	P
10	*petaurista sp.*	giant flying squirrel	LC	Sch II (Part I)	Pr (2,6)	-	J,N,L
11	*Callosciurus erythraeus*	red-bellied squirrel	LC		Pr (2,6)	-	J,N,L,R,P

12	*Callosciurus pygerythrus*	hoary-bellied squirrel	LC		Pr (2,6)	-	J,N,L,R,P
13	*Dremomys lokriah*	orange-bellied himalayan squirrel	LC		Pr (1)	-	R
14	*Rhizomys pruinosus*	hoary bamboo rat	LC	Sch V	Pr (4,6)	-	J,N,L,R,P
15	*Atherurus macrourus*	Asiatic brush-tailed porcupine	LC	Sch II (Part I)	Pr (6)	2018	J,N,L,R,P
16	*Hystrix brachyura*	himalayan crestless porcupine	LC		Pr (4,6)	-	J,N,L,R,P
	Lagomorpha						
17	*Lepus nigricollis*	Indian hare	LC	Sch IV	Pr (1,3,6)	-	J,N,L,R,P
	Pholidota						

18	*Manis pentadactyla*	Chinese pangolin	CR	Sch I (Part I)	Pr (4,6)	-	J,N,L,R,P
	Carnivora						
19	*Pardofelis marmorata*	marbled cat	NT	Sch I (Part I)	Pr (6)	-	J,N,L,P
20	*Prionailurus bengalensis*	leopard cat	LC	Sch I (Part I)	Pr (1,6)	-	J,N,L,R,P
21	*Prionailurus viverrinus*	fishing cat	VU	Sch I (Part I)	Pr (6)	-	N,L
22	*Neofelis nebulosa*	clouded leopard	VU	Sch I (Part I)	Pr (1,2,6)	-	J,N,L,R,P
23	*Panthera pardus*	leopard	VU	Sch I (Part I)	Pr (1,3,4,6)	-	J,N,L,R,P
24	*Paguma larvata*	masked palm civet	LC	Sch II (Part I)	Pr (1,2)	-	N,L,P
25	*Paradoxurus hermaphroditus*	Asian palm civet	LC	Sch II (Part I)	Pr (1,6)	-	J,N,L,R,P

26	*Viverra zibetha*	large Indian civet	LC	Sch II (Part I)	Pr (1)	-	P
27	*Viverricula indica*	small Indian civet	LC	Sch II (Part I)	Pr (1)	-	J,N,R,P
28	*Herpestes edwardsii*	Indian grey mongoose	LC	Sch II (Part I)	Pr (6)	-	P
29	*Herpestes urva*	crab-eating mongoose	LC	Sch II (Part I)	Pr (2,3,6)	-	J,N,L,R,P
30	*Vulpes bengalensis*	Indian fox	LC	Sch II (Part I)	Pr (6)	2017	J,N,L,R,P
31	*Melursus ursinus*	sloth bear	VU	Sch I (Part I)	Pr (6)	2017	J
32	*Ursus thibetanus*	Asiatic black bear	VU	Sch II (Part I)	Pr (6)	2007	J,N,L,R
33	*Lutrogale perspicillata*	smooth-coated otter	VU	Sch II (Part I)	Pr (2,6)	-	N,R,P

34	*Martes flavigula*	yellow-throated marten	LC	Sch II (Part I)	Pr (1,6)	-	J,N,L,R,P
35	*Mustela kathiah*	yellow-bellied weasel	LC	Sch II (Part I)	Pr (6)	2017	J,N,L,R
36	*Mustela strigidorsa*	stripe-backed weasel	LC		Pr (6)	2017	P
	Cetartiodactyla						
37	*Sus scrofa*	Indian wild boar	LC	Sch III	Pr (1,4,6)	-	J,N,L,R,P
38	*Muntiacus vaginalis*	barking deer	LC	Sch III	Pr (1,3,4,6)	-	J,N,L,R,P
39	*Rusa unicolor*	sambar	VU	Sch III	Pr (4)	-	J
40	*Capricornis rubidus*	red serow	NT		Pr (6)	2012	N,L,R,P

	Carnivora						
1	*Panthera tigris*	tiger	EN	Sch I (Part I)	Ab	1992	-

2	*Canis aureus*	golden jackal	LC	Sch II (Part I)	Ab	1979	-
3	*Cuon alpinus*	wild dog	EN	Sch II (Part I)	Ab	1979	-
	Cetartiodactyla						
4	*Axis porcinus*	hog deer	EN	Sch III	Ab	-	-
5	*Bubalus arnee*	wild water buffalo	EN	Sch I (Part I)	Ab	-	-
6	*Bos gaurus*	gaur	VU	Sch I (Part I)	Ab	-	-

[a-] LC = Least Concern; NT = Near threatened; VU = Vulnerable; EN = Endangered; CR = Critically Endangered;

[b-] Ab = Absent; Pr = Present; 1 = Camera trap; 2 = Direct sighting; 3 = Faecal deposit; 4 = Footprints/ burrow; 5 = Call; 6 = Local informant;

[c-] J = Jirang CR; N = Nongsangu CR; L = Lum Jusong CR; R = Raid Nongbri CR; P = Pdah Kyndeng CR
