# Supplementary material for: Community Reserves: Their significance for the conservation of mammals in a mosaic of community-managed lands in Meghalaya, Northeast India
Source: PLoS One. 2023 Jan 26;18(1):e0280994. doi: 10.1371/journal.pone.0280994 (PMC9879402; doi:10.1371/journal.pone.0280994)
Supplement: S3 Table — (PDF) [file pone.0280994.s005.pdf]

Community Reserves: their significance for conservation of mammals in a mosaic of community-managed lands in Meghalaya, Northeast India

Table S3. Perception of respondents to trends in mammal species population, abundances and their observation of wildlife

**Species**	**PKCR (n = 15)**	**LJ/NCR (n = 15)**	**RNCR (n = 30)**	**JCR (n = 15)**	**Log-likelihood ratio**	**P**
**Decrease in population**						
elephant	0.0	0.0	0.0	13.3	-3.2	0.12
Bengal slow loris	0.0	0.0	43.3	0.0	-11.9	**0.00**
capped langur	0.0	6.7	3.3	6.7	-0.8	1.00
hoolock gibbon	6.7	33.3	0.0	6.7	-5.7	**0.00**
flying squirrel	0.0	0.0	0.0	6.7	-1.6	0.60
crestless porcupine	6.7	0.0	0.0	0.0	-1.6	0.60
Chinese pangolin	60.0	20.0	23.3	6.7	-4.1	0.06
tiger	0.0	33.3	66.7	13.3	-10.0	**0.00**
mongoose	6.7	0.0	0.0	0.0	-1.6	0.60
bear	40.0	60.0	3.3	6.7	-9.0	**0.00**
yellow-throated marten	6.7	0.0	0.0	0.0	-1.6	0.60
wild boar	93.3	6.7	20.0	6.7	-10.9	**0.00**
barking deer	93.3	73.3	90.0	20.0	-5.0	**0.00**
sambar	6.7	13.3	0.0	0.0	-2.9	0.17

serow	6.7	0.0	0.0	0.0	-1.6	0.60
**Increase in population**						
elephant	0.0	0.0	0.0	33.3	-8.1	**0.00**
macaque	0.0	93.3	90.0	26.7	-13.9	**0.00**
capped langur	0.0	0.0	0.0	6.7	-1.6	0.60
hoolock gibbon	0.0	0.0	3.3	0.0	-0.9	1.00
crestless porcupine	0.0	20.0	10.0	6.7	-2.2	0.39
Chinese pangolin	0.0	6.7	0.0	0.0	-1.6	0.60
leopard cat	0.0	13.3	0.0	0.0	-3.2	0.12
leopard	0.0	0.0	46.7	0.0	-12.8	**0.00**
mongoose	0.0	0.0	3.3	0.0	-0.9	1.00
wild boar	0.0	0.0	0.0	33.3	-8.1	**0.00**
barking deer	0.0	0.0	0.0	13.3	-3.2	0.12
**No change in population**						
elephant	0.0	0.0	0.0	33.3	-8.0	**0.00**
Bengal slow loris	0.0	0.0	10.0	0.0	-2.8	0.23
macaque	0.0	0.0	0.0	26.7	-6.4	**0.01**
capped langur	0.0	6.7	0.0	6.7	-1.8	0.52
hoolock gibbon	0.0	26.7	16.7	6.7	-3.2	0.15
crestless porcupine	0.0	0.0	0.0	13.3	-3.2	0.12
Chinese pangolin	0.0	20.0	0.0	6.7	-4.2	**0.04**
leopard cat	0.0	6.7	0.0	0.0	-1.6	0.60
leopard	0.0	6.7	26.7	0.0	-5.8	**0.01**

tiger	0.0	0.0	3.3	0.0	-0.9	1.00
bear	0.0	0.0	0.0	6.7	-1.6	0.60
wild boar	0.0	0.0	0.0	20.0	-4.8	**0.02**
barking deer	0.0	0.0	3.3	20.0	-3.5	0.14
**Most observed**						
elephant	0.0	0.0	0.0	46.7	-11.3	**0.00**
Bengal slow loris	13.3	6.7	16.7	6.7	-0.7	0.75
macaque	80.0	100.0	96.7	73.3	-0.5	0.82
capped langur	0.0	20.0	0.0	20.0	-5.5	**0.02**
hoolock gibbon	20.0	53.3	46.7	46.7	-1.4	0.44
flying squirrel	0.0	20.0	0.0	0.0	-4.8	**0.02**
squirrel	26.7	40.0	30.0	20.0	-0.5	0.80
brush-tailed porcupine	0.0	6.7	0.0	0.0	-1.6	0.60
crestless porcupine	0.0	33.3	36.7	0.0	-8.2	**0.00**
hare	0.0	46.7	6.7	13.3	-6.3	**0.01**
Chinese pangolin	0.0	13.3	6.7	0.0	-2.3	0.31
leopard cat	13.3	40.0	56.7	0.0	-8.3	**0.00**
leopard	0.0	20.0	80.0	0.0	-17.4	**0.00**
tiger	0.0	0.0	13.3	0.0	-3.7	0.10
civet	26.7	0.0	0.0	0.0	-6.4	**0.00**
mongoose	80.0	13.3	33.3	0.0	-9.7	**0.00**
fox	0.0	13.3	10.0	0.0	-2.6	0.37
bear	0.0	0.0	3.3	0.0	-0.9	1.00

yellow-throated marten	0.0	0.0	0.0	13.3	-3.2	0.12
wild boar	0.0	13.3	20.0	60.0	-7.0	**0.00**
barking deer	26.7	33.3	26.7	26.7	-0.1	0.99
**Most abundant**						
elephant	0.0	0.0	0.0	73.3	-17.7	**0.00**
Bengal slow loris	0.0	0.0	0.0	6.7	-1.6	0.60
macaque	100.0	100.0	100.0	80.0	-0.3	0.92
capped langur	0.0	0.0	3.3	20.0	-3.5	0.14
hoolock gibbon	60.0	26.7	43.3	26.7	-1.5	0.43
flying squirrel	0.0	13.3	0.0	0.0	-3.2	0.12
squirrel	40.0	60.0	40.0	0.0	-6.5	**0.01**
brush-tailed porcupine	0.0	6.7	0.0	13.3	-2.9	0.17
crestless porcupine	0.0	60.0	50.0	20.0	-7.8	**0.00**
hare	6.7	20.0	3.3	33.3	-3.7	0.09
Chinese pangolin	0.0	6.7	0.0	6.7	-1.8	0.52
leopard cat	6.7	46.7	13.3	0.0	-5.9	**0.01**
leopard	0.0	6.7	76.7	0.0	-18.5	**0.00**
civet	40.0	6.7	0.0	6.7	-7.0	**0.00**
mongoose	40.0	0.0	6.7	6.7	-5.5	**0.02**
fox	0.0	20.0	13.3	0.0	-3.7	0.11
yellow-throated marten	0.0	0.0	0.0	6.7	-1.6	0.60
wild boar	0.0	0.0	0.0	80.0	-19.3	**0.00**
barking deer	0.0	6.7	0.0	26.7	-5.6	**0.01**

**Least observed**						
elephant	0.0	0.0	0.0	6.7	-1.6	0.60
Bengal slow loris	6.7	26.7	56.7	0.0	-9.3	**0.00**
macaque	6.7	0.0	0.0	0.0	-1.6	0.60
capped langur	0.0	33.3	16.7	0.0	-5.7	**0.01**
hoolock gibbon	0.0	6.7	0.0	13.3	-2.9	0.17
Chinese pangolin	0.0	13.3	63.3	0.0	-14.0	**0.00**
leopard cat	0.0	6.7	3.3	0.0	-1.1	1.00
leopard	0.0	6.7	6.7	0.0	-1.5	0.71
tiger	0.0	0.0	76.7	80.0	-17.9	**0.00**
mongoose	6.7	6.7	0.0	0.0	-1.8	0.52
fox	0.0	0.0	0.0	6.7	-1.6	0.60
bear	0.0	66.7	13.3	40.0	-8.8	**0.00**
wild boar	0.0	33.3	26.7	6.7	-4.7	**0.04**
barking deer	0.0	46.7	63.3	33.3	-7.9	**0.00**
sambar	0.0	0.0	0.0	46.7	-11.3	**0.00**
**Least abundant**						
Bengal slow loris	6.7	26.7	76.7	0.0	-13.5	**0.00**
capped langur	0.0	13.3	10.0	0.0	-2.6	0.37
hoolock gibbon	0.0	20.0	0.0	0.0	-4.8	**0.02**
crestless porcupine	6.7	0.0	0.0	0.0	-1.6	0.60
Chinese pangolin	53.3	46.7	50.0	0.0	-6.7	**0.01**
leopard cat	0.0	0.0	3.3	0.0	-0.9	1.00

leopard	0.0	0.0	13.3	0.0	-3.1	0.20
tiger	0.0	46.7	86.7	80.0	-11.3	**0.00**
mongoose	6.7	6.7	0.0	0.0	-1.8	0.52
fox	0.0	6.7	0.0	0.0	-1.6	0.60
bear	40.0	60.0	10.0	40.0	-4.9	**0.03**
yellow-throated marten	6.7	0.0	0.0	0.0	-1.6	0.60
wild boar	73.3	26.7	40.0	0.0	-7.9	**0.00**
barking deer	86.7	73.3	83.3	13.3	-5.8	**0.01**
sambar	6.7	13.3	0.0	46.7	-8.1	**0.00**
wild water buffalo	0.0	0.0	0.0	13.3	-3.2	0.12
serow	6.7	0.0	0.0	0.0	-1.6	0.60
goral	0.0	6.7	0.0	0.0	-1.6	0.60
