# Supplementary material for: Community Reserves: Their significance for the conservation of mammals in a mosaic of community-managed lands in Meghalaya, Northeast India
Source: PLoS One. 2023 Jan 26;18(1):e0280994. doi: 10.1371/journal.pone.0280994 (PMC9879402; doi:10.1371/journal.pone.0280994)
Supplement: S4 Table — ‘-‘: Indicates not recorded; highlighted figures indicate RAI values of present study higher than the median. (PDF) [file pone.0280994.s006.pdf]

Community Reserves: their significance for conservation of mammals in a mosaic of community-managed lands in Meghalaya, Northeast India

Table S4. Comparison of relative abundance index (RAI^{ct} – number of trap-days required to get a single photo capture of a species) derived from camera trap surveys for mammals in the Community reserves of Ri Bhoi, Meghalaya with nine other forests of south and southeast Asia

**Locations**	**CRs of Ri Bhoi district, Meghalaya, India**	**Hukaung valley, Myanmar**	**Kanchenjunga Landscape, Sikkim, India**	**Karen state, Myanmar**	**Khao Yai NP, Thailand**	**Kaziranga NP, Assam, India**	**Manas NP, Assam, India**	**Namdapha NP, Arunachal Pradesh, India**	**Median**
Reference	Present study (2018-2019)	Naing et al. (2015)	Sathyakumar et al. (2011)	Moo et al. (2018)	Jenks et al. (2011)	Rahmani et al. (2016)	Lahkar et al. (2018)	Datta et al. (2008)	

Type of camera trap	Passive	Passive	Passive	Passive	Passive	Passive	Passive	Passive	
Effort (number of trap-days)	1019	10750	6278	9511	6260	672	6173	1537	
Macaca sp.	93	60	43	105	169	34	-	37	60.0
murid sp.	40	-	-	-	313	-	-	-	**176.5**
orange-bellied himalayan squirrel	340	-	704	-	-	-	-	-	**522.0**
Indian hare	510	**-**	**-**	-	-	-	90	**-**	300.0

leopard cat	127	145	37	170	1565	224	31	-	**145.0**
clouded leopard	510	215	3061	528	783		187	768	**528.0**
leopard	127	10750	-	63	-	672	18	-	**127.0**
masked palm civet	204	1792	47	69	-	-	-	-	136.5
Asian palm civet	1019	189	-	53	3130	336	-	-	336.0
large Indian civet	1019	430	67	51	169	168	77	-	168.0

small Indian civet	510	977	-	-	-	112	37	-	311
yellow-throated marten	170	347	31	144	-	-	772	-	**170**
wild boar	255	103	141	13	104	14	18	512	103.5
barking deer	64	20	20	11	104	61	24	22	23

'-': Indicates not recorded; highlighted figures indicate RAI values of present study higher than the median
